# Detecting and Preventing Defensive Reactions Toward Persuasive Information on Fruit and Vegetable Consumption Using Induced Eye Movements

**DOI:** 10.3389/fpsyg.2020.578287

**Published:** 2021-01-11

**Authors:** Arie Dijkstra, Sarah P. Elbert

**Affiliations:** Faculty of Behavioural and Social Sciences, Department of Psychology, University of Groningen, Groningen, Netherlands

**Keywords:** eye movements, working memory, persuasion, emotion regulation, gender differences

## Abstract

**Objective:** Persuasive messages regarding fruit and vegetable consumption often meet defensive reactions from recipients, which may lower message effectiveness. Individual differences in emotion regulation and gender are expected to predict these reactions. In the working memory account of persuasion, inducing voluntary eye movements during the processing of the auditory persuasive information might prevent defensiveness and thereby increase message effectiveness.

**Methods:** Participants in two independently recruited samples from the general population (*n* = 118 and *n* = 99) listened to a negatively framed auditory persuasive message advocating fruit and vegetable consumption. Half of them were asked to keep following a regularly moving stimulus on their screen with their eyes. At pretest, the individual differences of cognitive self-affirmation inclination (CSAI) and gender were assessed to predict defensive reactions.

**Results:** In Study 1, induced eye movements significantly increased self-reported consumption after 2 weeks when CSAI was low, but only in males, as indicated by a significant three-way interaction (*p* < 0.001). With negative self-evaluative emotions as dependent variable, this three-way interaction was also significant (*p* < 0.05), suggesting that induced eye movements prevented defensiveness in low CSAI males. Study 2 did not assess consumption but replicated the latter three-way interaction (*p* < 0.05).

**Conclusion:** The studies replicated our earlier findings regarding the moderating effects of individual differences in emotion regulation (i.e., CSAI) on persuasion, but they also revealed gender differences in persuasion that are related to the working memory. The working memory account of persuasion provides new theoretical as well as practical angles on persuasion to target individuals in persuasion to increase fruit and vegetable consumption.

## Introduction

In the framework of health promotion, the proven relationship between fruit and vegetable consumption on the one hand, and cardiovascular diseases, cancer, and all-cause mortality on the other hand (Aune et al., 2013), might be translated into health messages directed at the general public. However, the effectiveness of health messages is often lowered by recipients’ defensive reactions, such as denial, biased processing, and message rejection (Liberman and Chaiken, 1992; Ruiter et al., 2001; Good and Abraham, 2007). Also with regard to messages and interventions that advocate fruit and vegetable consumption, several studies suggest or identify such detrimental reactions (Epton and Harris, 2008; Dijkstra et al., 2011; Pietersma and Dijkstra, 2011b; Thompson and Kumar, 2011; Elbert and Dijkstra, 2014; Ungar et al., 2015; Fielden et al., 2016; van Koningsbruggen et al., 2016). As these defensive reactions can lower the effectiveness of persuasive messages regarding fruit and vegetable consumption, they may be partly responsible for the high proportion of people that does not consume fruit and vegetables according the guidelines (Lee-Kwan et al., 2017; Eurostat Statistics Explained, 2020). Therefore, understanding such detrimental psychological reactions toward health messages is one important direction for research in health promotion and persuasion regarding fruit and vegetable consumption.

### The Working Memory Account

In the present theorizing, persuasive processes take place in the working memory (WM). The WM is the virtual place where attention is directed, where incoming information is compared to stored information and where ongoing reactions are initiated and regulated [(Baddeley, 1986, 2012; Diamond, 2013) for a different conceptualization]. The WM account of persuasion assumes that persuasive processes take place in the WM and that two phases can be recognized.

In the first phase, the persuasive information enters the WM, where it is linked to information from the long-term memory (Symons and Johnson, 1997; Kruglanski and Thompson, 1999); activated long-term memory contents may then give self-relevant meaning to the incoming information. This meaningful information that is now held in the WM may be represented in a more or less vivid mental image (Kosslyn et al., 1983; Pearson et al., 2015). In persuasion, this mental image will be about the persuasive outcomes in a message (Bruyer and Scailquin, 1998; Bolls and Lang, 2003), for example, the negative consequences of consuming a low level of fruit and vegetables. As the mental image is compared to a standard (related to values and goals), it may trigger the experience of a threat, possibly with its accompanying emotions, such as fear (Witte, 1992), and negative self-evaluative emotions (Dijkstra and Buunk, 2008). These are aversive experiences that people are motivated to avert.

When the threat passes a certain threshold, the second phase may be activated to down-regulate the threat—the defensive reactions are mobilized to lower the aversive feelings of threat caused by the persuasive message, in health behavior especially negative self-evaluative emotions. One emerging perspective on defensive reactions is that they also can be “located” in the WM, as they can be conceptualized as self-regulatory actions (Baumeister and Vonasch, 2015; Dijkstra, 2018) or as a manifestation of emotion regulation (Koole and Aldao, 2005; Gross, 2007). These cognitive self-regulatory actions may consist of processes that reject the persuasive message or processes that direct the behavior toward a solution in line with the persuasive message (Witte, 1992).

Thus, the WM account of persuasion assumes that the development of mental images (Bruyer and Scailquin, 1998; Bolls and Lang, 2003; Gunter and Bodner, 2008; Hout et al., 2011b) and the self-regulatory actions in response to the persuasive information (Hinson et al., 2003; Barrett et al., 2004) take place in the WM. A central premise is that the development of mental images and of self-regulatory actions is not for free: it needs WM space. This implies that when there is not enough WM space, one or both processes may not completely unfold. Mental images may fail to reach high quality (e.g., vividness), and/or self-regulatory actions may be prevented or disturbed, and not or less effective. This may have various effects on persuasion. Thus, the available WM space can be expected to influence persuasion, which implies that, taxing the WM with another, competing task will influence persuasion. One way to tax the WM is by inducing regular eye movements. In persuasion, induced regular eye movements can tax the WM while the persuasive information is presented through the auditory channel.

### Induced Eye Movements in Persuasion

Induced horizontal eye movements (EMi) have been studied in the context of understanding and treating posttraumatic stress disorder (Shapiro, 1999), in which fearful and traumatic memories are central (Davidson and Parker, 2001). In this approach, induced eye movements are a core element in the treatment referred to as Eye Movement Desensitization and Reprocessing. In this context, EMi have been shown to lower the vividness and emotionality of mental of images from autobiographical memories of negative past events (Gunter and Bodner, 2008; Hout et al., 2011a,b). However, in persuasion, the mental images are not about past events but about outcomes in the future (i.e., the effects of consuming a low level of fruit and vegetables). These mental images might be conceptualized as “distressing images about feared future events,” the so-called “flashforwards,” which also have been shown to become less vivid and less emotional after EMi (Engelhard et al., 2010). All these effects, including those in our own studies on persuasion (Dijkstra and Asten, 2013; Dijkstra and Elbert, 2019), seem to be best explained by a WM account in which the EMi demand WM space as a competing task.

As proposed above, in persuasion, mental images and self-regulatory reactions to these mental images are brought about in the WM. First, EMi can disturb the development of mental images of the persuasive outcomes. This can have two effects: either there is no persuasive power left, or there is some persuasive power left but the level of threat stays below a threshold, thereby preventing the mobilization of self-regulatory actions. In the latter case, EMi influence self-regulatory reactions indirectly (through effects on mental images). Second, EMi can disturb the development of self-regulatory reactions directly. The persuasive effect of disturbing the self-regulatory reactions will depend on the type of reaction—unfavorable or negative reactions or thoughts in persuasion have been shown to manifest as counterarguments that need self-regulatory resources and thus can be conceptualized as self-regulation (Wheeler et al., 2007; Dijkstra, 2018). When people react with defensive self-regulatory processes (negative/unfavorable thoughts), which have the potential to lower persuasion, EMi will disturb these inhibiting processes and therefore will lead to increased persuasion. However, people can also show favorable or positive reactions or thoughts, which can be conceptualized as self-regulation as well, and these also have been located in the WM (Kane and Engle, 2003; Hofmann et al., 2008). These reactions support persuasion, and disturbing these reactions with EMi will lower persuasion, an undesired side effect of EMi in practice. Therefore, it is important to predict who will react defensively and who will react positively. The individual difference “cognitive self-affirmation inclination” (CSAI) has been shown to predict negative as well as positive reactions toward threatening persuasive information (Pietersma and Dijkstra, 2011a; Dijkstra and Elbert, 2019).

**FIGURE 1:**
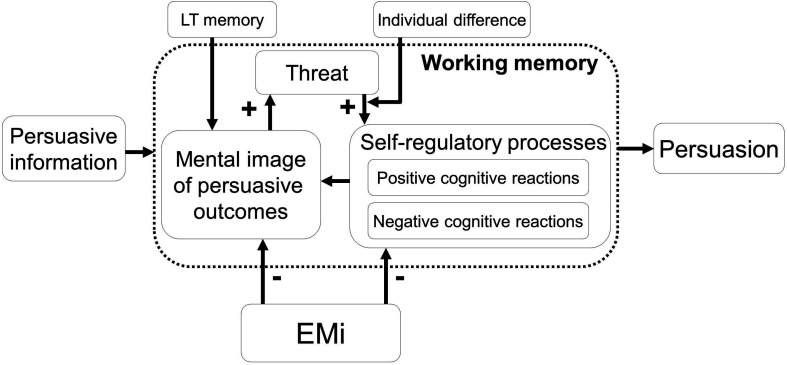
The working memory account of persuasion and induced eye movements.

The CSAI is an individual difference measure of emotion regulation. A high score on the measure of CSAI indicates a strong inclination to cope with a self-threat by thinking of compensating positive self-images, selectively and functionally derived from one’s long-term memory. Similar to the effect of a self-affirmation procedure (Harris and Napper, 2005; Sweeney and Moyer, 2015), these people process the persuasive information open-minded and become painfully aware of their own role in generating unhealthy effects, leading to negative self-evaluation (Jessop et al., 2009; van Koningsbruggen et al., 2016) and thereby to more persuasion (meant to lower these negative emotions). On the other hand, a low score on CSAI indicates the use of other strategies to deal with the threat. Our studies suggest that the intention of people with low CSAI was actively lowered by defensive self-regulatory actions (Pietersma and Dijkstra, 2011a). Those low in CSAI are expected to hold off the threat by negative cognitive reactions during the processing of the information, which inhibits persuasion. Figure 1 summarizes the main concepts in the present WM account of persuasion (WMAP; 39).

In a recent study, these predictions from the WMAP were verified (Dijkstra and Elbert, 2019). In people scoring low on CSAI on pretest, EMi significantly increased fruit and vegetable consumption, whereas in people scoring high on CSAI on pretest, EMi significantly lowered fruit and vegetable consumption. This means that we can now predict who will react defensively to a message promoting fruit and vegetable consumption and in whom EMi can have beneficial effects. The present study aims not only to replicate these findings but also to bring this line of research one step further.

One issue concerns the threat that is caused by vivid mental images. In a behavior that is in principle under control of the individual, learning about the negative consequences of one’s own behavior will lead to negative self-evaluative emotions. We have shown that these emotions are consistent and powerful predictors of behavior change (Dijkstra et al., 1999; Dijkstra and Dijker Den, 2005; Dijkstra and Buunk, 2008). It is expected that people high in CSAI will experience a strong negative self-evaluation when they process a health message (they are open-minded and acknowledge their own role), but EMi will lower this as the mental images that lead to the threat, and the subsequent emotions are prevented to fully unfold. In contrast, people who are low in CSAI will experience less negative self-evaluation when they process a health message because they apply defensive self-regulation to avert the negative emotions. This defensive regulation leads to peace of mind. EMi will disturb the defensive self-regulation, thereby leaving the mental images and their subsequent threat and negative emotions to fully unfold and get their persuasive momentum.

During the development of our expectation on the basis of the WMAP, we ran into a variable that seemed to be related inherently to WMAP’s two main processes: (1) experiencing negative emotions in reaction to external information and (2) related emotion regulation: gender. There are some studies that show gender differences in emotional reactions toward potentially threatening information, with males experiencing stronger negative affective reactions toward such messages, including aggression, although overall the results are mixed (McRae et al., 2008). For example, a meta-analysis concluded that men probably are “more easily aroused by [aggressive relevant] emotionally evocative stimuli” (p. 379; 50). However, with regard to gender differences in persuasion, the results seem more consistent. There are several reports that suggest that males react more defensively toward persuasive attempts: Males were more likely than females to perceive negative affect and source derogation to be helpful strategies for resisting persuasion (Zuwerink Jacks and Cameron, 2003); males showed a stronger “third-person effect,” meaning that they rated messages more influential for other people than for themselves (Lewis et al., 2007); males scored higher on a measure of message derogation (Kamboj et al., 2016), and they more strongly downgraded the problem that was communicated in the persuasive messages (Goldenbeld et al., 2008). This gender difference in defensiveness is backed up by literature on emotion regulation (Diehl et al., 1996). For example, in contrast to females, males showed a tendency to deny depressive symptoms (Joiner et al., 2000); males used less rumination, reappraisal, and acceptance (Nolen-Hoeksema and Aldao, 2011), and differences in the neural bases of emotion regulation are observed (Williams et al., 2005; McRae et al., 2008; Whittle et al., 2011). In summary, there is evidence that males and females can differ in reactions toward persuasive messages, with males being more defensive. In our theorizing, this means that males will be less persuaded by our health message than females, but that EMi will disturb these defensive reactions, leading to more persuasion and to stronger negative self-evaluative emotions.

### The Present Studies

The global aim is to study the effects of EMi in persuasion. In two EMi versus no EMi online experiments in the general population, participants listen to a negatively framed persuasive audio message promoting fruit and vegetable consumption, whereas in half of them EM will be induced (following a stimulus on a computer screen with one’s eyes). In Study 1, the primary dependent variable is the actual (self-reported) fruit and vegetable consumption assessed after 2 weeks. In Studies 1 and 2, the effects of EMi on negative self-evaluation assessed immediately after the message exposure will be assessed.

Because EMi can only influence the persuasive process in the desired direction when recipients are defensive, EMi is expected to lead to more fruit and vegetable consumption in those who score low on CSAI. Here we aim to replicate our earlier study (Dijkstra and Elbert, 2019). Expanding the former study, EMi is also expected to lead to a more negative self-evaluation in those who score low on CSAI. These effects depend on the defensiveness of people with low CSAI, but the same is expected for males. Because especially males are expected to be defensive toward persuasive attempts, EMi will increase their consumption and their negative self-evaluation. Lastly, it will also be explored whether both moderators work synergistically and whether the combination of low CSAI and male gender is related to the behavioral and psychological effects of EMi.

## Study 1

### Methods

#### Recruitment

Participants were recruited in the Netherlands and in Germany in, initially, two online experiments. The aim to include at least 50 participants per condition was reached by combining the data from both experiments. In both countries, the participants were recruited through Facebook, to reach a population with variance in gender, age, and education level. The call to join a study of the University of Groningen on fruit and vegetable consumption was published on more than 40 Facebook pages, during a period of approximately 5 weeks.

#### Design

All participants listened to a negatively framed auditory health message on fruit and vegetable consumption, while they were randomly assigned to one of two conditions: induced eye movements (EMi) or no induced eye movements (no EMi). The process measure, negative self-evaluative emotions, was assessed immediately after the manipulations. The outcome measure self-reported fruit and vegetable consumption was assessed after 2 weeks. The study was approved by the Ethical Committee Psychology of the Faculty of Behavioral and Social Sciences.

#### Procedure

Participants who followed the link in the call entered the Qualtrics system in which they were welcomed and were provided with information on the coming study. They were informed that they would be asked to answer some personal questions and then listen to an auditory message on the negative consequences of low fruit and vegetable consumption and answer some remaining questions. In addition, they were told that they would receive a link to another brief questionnaire after 2 weeks and that they could join a lottery for an amount of 50 euros when they would fill in their email address. Besides further formal and legal information on research ethics, data storage, and privacy, they were asked to join the study using a device with a large screen, not on their smartphone. Lastly, they were asked for their formal consent to join the study by clicking the “proceed” button.

Demographics and several brief measures of individual differences were assessed before participants entered the conditions. After being assigned randomly to one of both conditions, participants were instructed for the specific manipulations, and next, they were exposed to the manipulations. All participants were asked to listen to the auditory persuasive message, while half of them were instructed to keep watching the moving stimulus (see later). After that, they were asked some questions concerning the process measures, and they were thanked and invited to join the study in 2 weeks. Participants who gave their email address were sent a link to the follow-up questionnaire after 2 weeks. After self-reporting their fruit and vegetable consumption, they were debriefed.

#### The Persuasive Message

The auditory persuasive message (Dijkstra and Asten, 2013) advocated fruit and vegetable consumption and was negatively framed. It comprised approximately 240 words (110 s) that mentioned the possible negative outcomes of not eating sufficient levels of fruit and vegetables (mainly losses, e.g., “larger risk for cancer”).

Besides the information on these major outcomes related to low fruit and vegetable consumption, consumption was said to be related to looking less healthy, to worsened physical stamina, and to aging (“unhealthier skin and hair”). Two intermediary physical states were presented to be related to these consequences: “high blood pressure” and “high cholesterol.” These effects were said to be related to lowered intake of vitamins C and E.

To be able to induce eye movements (using a stimulus on the screen), the message was offered through the auditory channel. Female voices that presented the message (in Dutch and in German, respectively) were carefully selected and recorded in professional recording studios. The actor was instructed to speak at a normal rate, with normal intonation, as the actor would read it like a professional newsreader.

#### The EMi Manipulation

In the EMi condition, participants were instructed to listen to the auditory message and at the same time look at the moving stimulus. In the German study, the stimulus was a black dot moving on a gray screen. For technical reasons, the stimulus in the Dutch study was altered into a red square on a white screen. The instructions were the same: “On the screen you will see a dot/square moving from left to right and vice versa. Please, follow the dot/square with your eyes all the time while listening to the auditory message.” On a 30-cm screen, the size of the dot/square was 20 mm. It moved from one side of the screen to the other in 2 s (independent of screen size), and it kept on moving until the auditory message was finished. Participants in the no-EMi condition were not presented with a moving dot/square and listened only to the audio message that was accompanied by a (gray or white) stable screen.

#### Measures

##### Pretest measures

In the first part of the questionnaire, participants were asked for their gender and age. In addition, they were asked to provide a judgment on their fruit and vegetable consumption: They could finish the sentence, “In general, I eat…,” which was followed by five options they had to choose one from: “far too little fruit” (1), “too little fruit” (2), “somewhat too little fruit” (3), “sufficient fruit” (4), “more than sufficient fruit” (5). The same format was used to assess perceived vegetable intake. The scores on both items were averaged to form a score of “discrepancy,” with a lower score representing a large discrepancy from one’s standard. The correlation between the fruit item and the vegetable item was *r* = 0.45, *p* < 0.001.

Cognitive self-affirmation inclination was assessed with six items on the experienced frequency of having specific self-related positive thoughts (Pietersma and Dijkstra, 2011a). Our earlier study showed that high scores on the CSAI led to the open-minded processing of threatening information, similar to the effects of a self-affirmation procedure. Low scores led to a defensive reaction toward a (moderate) threat. The following statements were part of the CSAI scale: “I notice that I do some things very well;” “When I feel bad about myself, I think about all the things that I can be proud of;” “I think about the things I in the past I did well;” “I think about all the things that I have successfully accomplished;” “When I have done something wrong that makes me feel dissatisfied with myself, I tell myself that I do not do everything wrong;” and “I realize that besides all the ‘stupid’ things I do, I also do some things very well.” These items could be scored on a perceived frequency scale: “never” (1), “sometimes” (2), “regularly” (3), “often (4), “very often” (5). The Cronbach α was 0.78.

Some other individual difference measures were applied that will not be presented here, as they are not pertinent to the present study.

##### Posttest measures immediate

After listening to the message, participants in the EMi condition were asked how well they succeeded in watching the dot/square: “To what extend did you succeed in watching the dot/square all the time?” This question could be answered on a scale ranging from “not at all” (1) to “completely” (seven in the Dutch study, nine in the German study). Next, all participants were asked how well they succeeded in listening to the audio text. (Only in the Dutch study the first questions after this part were about the quality of the mental images participants experienced during the exposure. These data will not be analyzed in the present study). A one-item measure of negative self-evaluative emotions was applied (Dijkstra and Buunk, 2008): “After having listened to the text, how dissatisfied are you with yourself?” This item could be answered from “not at all dissatisfied” (1) to “very dissatisfied” (7). Lastly, one item on fear and a four items on intention were applied, but not used for the present study.

##### Posttest measure follow-up

Two weeks after completion of the experiment, respondents were sent the link to the online follow-up questionnaire as part of the study. The questionnaire was a detailed and validated self-report frequency questionnaire on the own average weekly fruit and vegetable intake during the past 2 weeks (Bogers et al., 2004; Elbert et al., 2016). Respondents were asked to indicate how often on average they ate or drank products from several fruit and vegetable categories during the previous weeks per week. The answering options ranged from “never or less than 1 day a week” (0), “1 day a week” (1) to “every day” (7). Next, they were asked to indicate the amount of intake per category of fruit or vegetables in terms of pieces of fruit and servings of vegetables (answering options ranged from “no pieces/glasses/serving spoons” to “five or more pieces/glasses/serving spoons”). The main categories were “cooked vegetables,” “raw vegetables/salad,” “fruit/vegetable juice,” “tangerines,” “oranges/grapefruits/lemons,” “apples/pears,” “bananas,” “other fruit,” and “apple sauce.” The average number of days per week and the pieces of fruit and vegetable portions (defined as 50 g each) were multiplied for each category and added to create a composite index of weekly fruit and vegetable intake.

### Results

#### Selection and Attrition Analyses

The main analyses on our two outcome measures, negative self-evaluative emotions (assessed immediately after the manipulations) and self-reported fruit and vegetable consumption (assessed after 2 weeks), were conducted in the cohort with complete 2-week follow-up data. This cohort was composed by the following attrition and selection procedure. In total, 590 participants entered the system and gave their consent by clicking the button, and 70 immediately stopped after that. Of the remaining 520 participants, 372 got to the page on which the audio procedure was introduced; they were randomized to one of both conditions. Of these 372 participants, another 44 did not provide immediate posttest data, leading to 328 participants. Of the 167 participants in the EMi condition, 18 scored under the midpoint on the scale asking to what extent they succeeded in “keeping their eyes on the dot/square all the time.” These were excluded from the analyses, leaving 149 participants in the EMi condition and 161 in the no-EMi condition. Of these 310 participants, 118 (38%) provided complete follow-up data (57 in the EMi condition, 61 in the no-EMi condition). Of these participants in whom the main analyses were run, 33.1% (*n* = 39) had joined the study on their smartphone.

To check whether a relevant selection had occurred from the participants who gave their consent and entered the study system, these 118 in the final cohort were compared to the 340 participants who provided no follow-up data but of whom the following measures were available: age, gender, CSAI, discrepancy, and pretest intention. No significant differences were found between these groups on these variables, although with regard to discrepancy the test approached significance (*p* = 0.067), suggesting a stronger discrepancy in the participants who were excluded. The experimental conditions also did not differ significantly on the proportions of participants who provided follow-up data.

#### Sample Characteristics and Randomization Check

The sample of 118 participants consisted of 66.6% females (one missing value on gender); the average age was 32.6 years [standard deviation (SD) = 16.35 years; one missing value on age]; the mean score on CSAI was 2.92 (SD = 0.67); the mean discrepancy score was 3.24 (SD = 0.87), and pretest intention was on average 3.76 (SD = 1.50). To check the randomization, the EMi conditions were compared on these five variables. The conditions did not differ significantly (*p*’s > 0.31) on all these variables, suggesting that the randomization was successful.

#### Relations Among the Main Variables

To investigate the need to use pretest intention and discrepancy as covariates in the coming analyses of variance, correlations were computed. Pretest intention was significantly related to the dependent variable–negative self-evaluation, *r*(118) = 0.320, *p* < 0.001, not to fruit and vegetable consumption. Discrepancy was related significantly to both dependent variables: negative self-evaluation *r*(118) = −0.29, *p* < 0.01; self-reported consumption, *r*(118) = 0.319, *p* < 0.001. It was therefore decided to include pretest intention and discrepancy in all coming analyses as covariates. In addition, it was tested whether the two potential moderators, CSAI and gender, were related to each other and to both covariates. All these relations were not significant (*p*’s > 0.11).

#### CSAI and Gender as Independent Moderators

First, the moderating effects of CSAI and gender were tested separately. To start with, the expected two-way interaction between EMi and CSAI, including pretest intention and discrepancy as covariates, was tested for both dependent variables, negative self-evaluation and fruit and vegetable consumption. Both interactions approached significance, *F*(1,112) = 2.96, *p* = 0.088, η^2^ = 0.026, and *F*(1,112) = 3.14, *p* = 0.079, η^2^ = 0.054, respectively. The means for both dependent variables showed the expected pattern. In case of low CSAI (indicating defensive self-regulation), EMi was associated with higher means (disturbing the defensive self-regulation). In case of high CSAI (indicating supporting processes), EMi was associated with lowered means (disturbing supporting processes).

To test whether the main effect of EMi was significant when CSAI was low, and when it was high, the complete dataset (*n* = 118) was modeled to represent two levels of CSAI scores, by subtracting and adding one from the individual standardized scores (*z* scores), respectively (Cohen et al., 2003; Siero et al., 2009). However, for both dependent variables, the main effects of EMi in high and low CSAI were not significant (*p*’s > 0.11).

Next, the expected two-way interaction between EMi and gender, including pretest intention and discrepancy as covariates, was also tested for both dependent variables. Both interactions were not significant, *p*’s > 0.16. The mean scores on both variables were in the expected directions, with EMi being associated with more negative self-evaluation and a higher consumption, only in men. The main effects of EMi in females and males were not significant.

To conduct a last check for these two-way interactions, both interactions were tested in one model for each dependent variable (Yzerbyt et al., 2004). With self-evaluation as dependent variable, this model showed that both interactions approached significance (*p*’s > 0.09 < 0.10). With consumption as dependent variable, this model showed that only the CSAI × EMi interaction approached significance (*p* = 0.057). In summary, with regard to negative self-evaluation and fruit and vegetable consumption, the patterns of means related to both moderators, CSAI and gender, were all in expected directions (Dijkstra and Elbert, 2019) but the moderation effects at the best approached significance. Therefore, it was tested whether both moderators worked synergistically, in a three-way interaction.

#### The Three-Way Interaction

**FIGURE 2:**
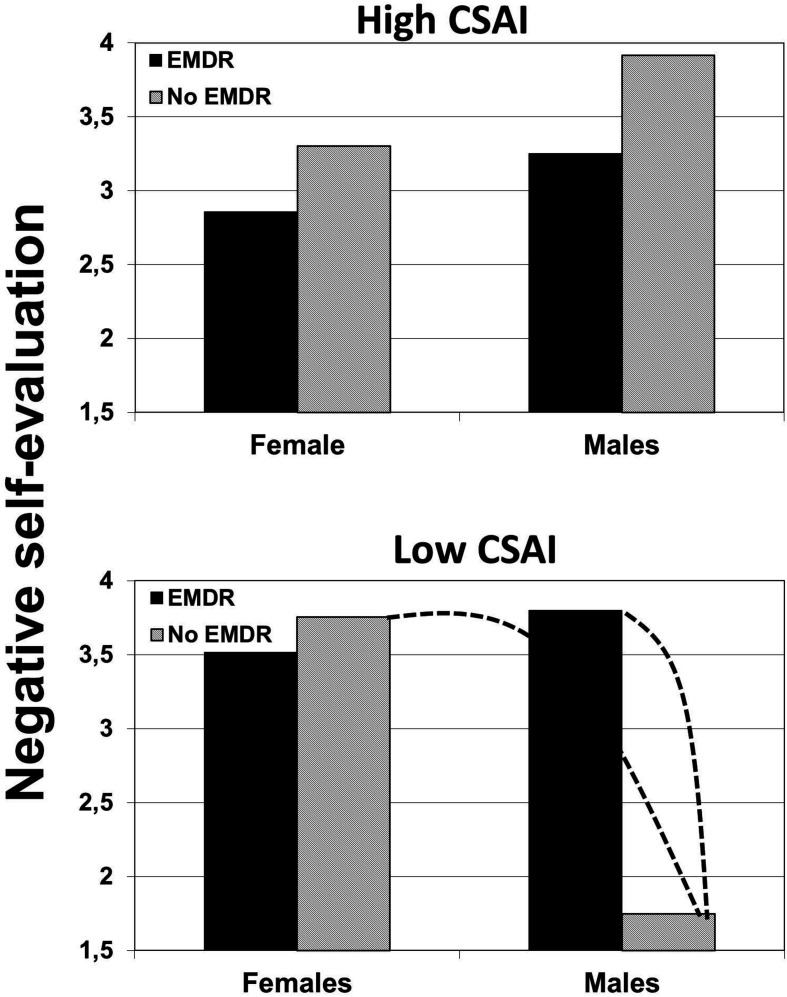
The effects of induced eye movements (EMi) on negative self-evaluation, moderated by CSAI and gender (the dotted line indicates a significant difference).

**FIGURE 3:**
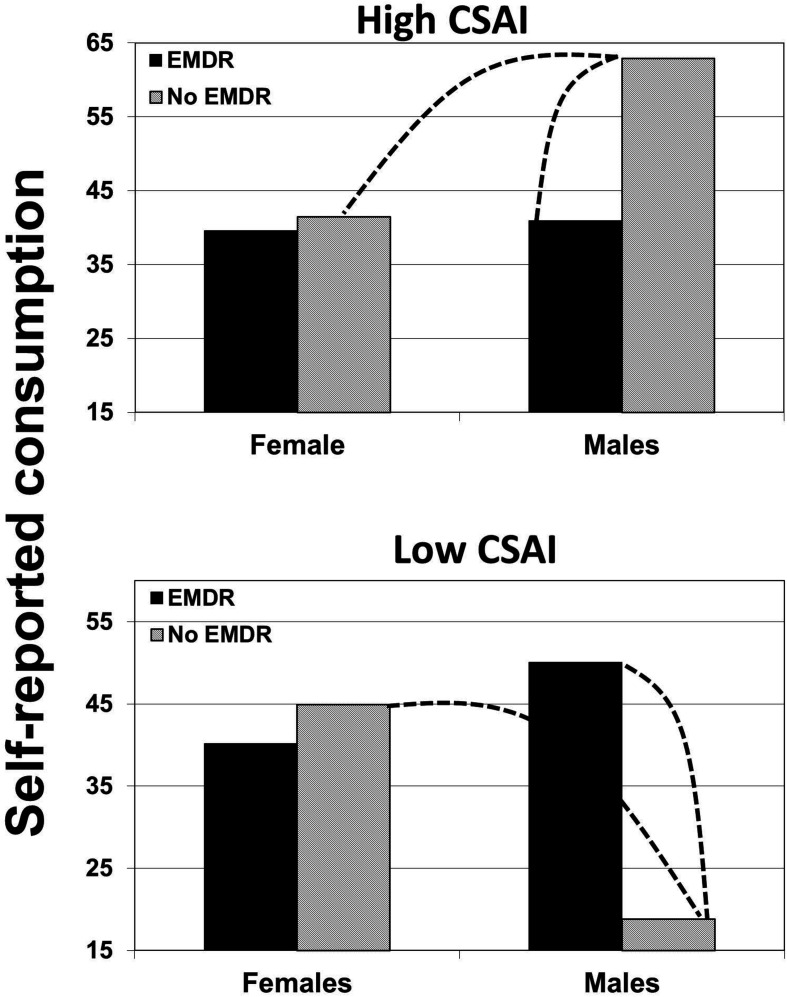
The effects of induced eye movements (EMi) on self-reported fruit and vegetable consumption, moderated by CSAI and gender (the dotted line indicates a significant difference).

For both dependent variables, a three-way interaction was conducted in a saturated model (with the three-way interaction as highest order factor), with pretest intention and pretest discrepancy as covariates, using an analysis of covariance (ANCOVA). With regard to negative self-evaluation, this three-way interaction was significant, *F*(1,107) = 4.41, *p* < 0.038, η^2^ = 0.04, as it was for self-reported fruit and vegetable consumption, *F*(1,107) = 12.99, *p* < 0.001, η^2^ = 0.10. Figures 2, 3 show the estimated means of the effects of EMi on self-evaluation and fruit and vegetable consumption, according to CSAI and gender.

#### Effects on Self-Evaluation

To further understand the significant three-way interaction, it was tested whether the interaction between gender and EMi differed within each of the two levels of CSAI. Using the same method as explained above, two levels of CSAI were modeled. When CSAI was modeled as low, the gender × EMi interaction was significant, *F*(1,107) = 6.78, *p* = 0.011, η^2^ = 0.04. As can be observed in Figure 2, the expected effect of EMi occurred among males. To further test the expected pattern, the interaction between CSAI and EMi in only males was tested, which was significant, *F*(1,33) = 8.84, *p* = 0.005, η^2^ = 0.21. Contrast analyses showed that in males, EMi (mean = 3.8) led to a significantly higher negative self-evaluation (mean = 1.75), *p* = 0.007, diff. 95% confidence interval (CI) = −3.53 to −0.57, η^2^ = 0.066. When CSAI was modeled as high, the gender × EMi interaction was not significant, *p* > 0.80, and no contrasts were significant.

To further test the observed effects, the interaction between gender and CSAI was analyzed when people listened only to the persuasive audio, thus without eye movement induction. This two-way interaction was significant, *F*(1,54) = 7.94, *p* = 0.005, η^2^ = 0.13, showing that the reactions of males and females to the persuasive audio depended on their CSAI. When CSAI was low, the mean self-evaluation of males (mean = 1.75) was significantly lower than the self-evaluation of females (mean = 3.75), *p* = 0.003, diff. 95% CI = −3.29 to −0.71, η^2^ = 0.081. Within the condition with the eye movement induction, the interaction was not significant, *F*(1,51) < 1, *p* = 0.32, η^2^ = 0.019.

#### Effects on Consumption

To further understand the significant three-way interaction with regard to self-reported fruit and vegetable consumption, it was tested whether the interaction between gender and EMi differed within each of the two levels of CSAI. Using the same method as explained above, two levels of CSAI were modeled. When CSAI was modeled as low, the gender × EMi interaction was significant, *F*(1,107) = 9.85, *p* = 0.002, η^2^ = 0.084. When CSAI was modeled as high, the gender × EMi interaction approached significance, *F*(1,107) = 3.71, *p* = 0.057, η^2^ = 0.033. As can be observed in Figure 3, the expected pattern was present in males, not in females. To test the expected pattern, the interaction between CSAI and EMi in only males was tested, which was significant, *F*(1,33) = 12.43, *p* = 0.001, η^2^ = 0.27. Contrast analyses within males showed that when CSAI was modeled as low, EMi (mean = 50.06) led to a significantly higher self-reported fruit and vegetable consumption compared to no EMi (mean = 18.81), *p* = 0.002, diff. 95% CI = −50.61 to −11.89, η^2^ = 0.087. When CSAI was modeled as high, EMi (mean = 40.9) led to a significantly lower self-reported fruit and vegetable consumption compared to no EMi (mean = 62.9), *p* = 0.008, diff. 95% CI = 5.98 to 37.99, η^2^ = 0.065.

To further test the observed effects, the interaction between gender and CSAI was analyzed when people listened only to the persuasive audio, thus without eye movement induction. This two-way interaction was significant, *F*(1,54) = 15.31, *p* < 0.001, η^2^ = 0.22, showing that the reactions of males and females to the persuasive audio depended on their CSAI. Contrast analyses showed that when CSAI was low, males reported a significantly lower fruit and vegetable consumption (mean = 18.81) compared to females (mean = 44.93), *p* = 0.003, diff. 95% CI = −42.97 to −9.26, η^2^ = 0.081. When CSAI was high, males reported a significantly higher fruit and vegetable consumption (mean = 62.9) compared to females (mean = 41.44), *p* = 0.011, diff. 95% CI = 5.0 to 37.9, η^2^ = 0.059.

#### *Post hoc* Analyses

To test whether negative self-evaluation mediated the effects of the CSAI × gender × Emi interaction on fruit and vegetable consumption, two tests were conducted. First, the data showed a non-significant correlation between negative self-evaluation and consumption, *r*(118) = −0.049, *p* ≥ 0.60. Second, the three-way interaction with consumption as DV was rerun, now with negative self-evaluation in the model as independent variable. As could be expected on the basis of the former analysis, only minor changes in the statistics of the three-way interaction occurred (e.g., the *F*-value changed from 12.99 to 12.73). Therefore, it can be concluded that there is no mediation, and no further testing of mediation was conducted.

Additional analyses were also conducted to rule out that the findings were caused by underlying differences between the experiments in the Netherlands and Germany, and between participants who joined the study on their smartphone and those who used a larger screen. The above saturated models with three-way interactions were expanded by now including also the three-way interaction of EMi, CSAI, and screen or the three-way interaction of EMi, CSAI, and country. None of the interaction tests was significant (*p*’s > 0.21), and only minor changes in *p*-values regarding negative self-evaluation and consumption occurred, suggesting no confounding of the core results by screen and country.

## Study 2

The aim of this study was only to replicate Study 1 with regard to negative self-evaluation: It is expected that the effect of EMi on negative self-evaluation is moderated by CSAI and gender. Especially in males with low CSAI, the negative self-evaluation will be low (indicating defensive self-regulation), and EMi will lead to a significant increase in negative self-evaluation.

### Method

The recruitment, the procedure, the persuasive message, the EMi procedure, and the immediate measures were all the same as in Study 1. There were two differences with Study 1. First, no follow-up data on fruit and vegetable consumption were available; the response at follow-up was only 20%, with very low counts in the EMi condition. Second, originally Study 2 had a 2 × 2 design; besides EMi, another manipulation was conducted (half of the participants received a forewarning of persuasive intent), but this had no detectable effects. Because this manipulation that was used for the first time seemed to be inert, it was ignored in the below analyses.

### Results

#### Sample Characteristics and Randomization Check

The sample of 99 participants consisted of 59.6% females, the average age was 34.7 years (SD = 13.9 years), the mean score on CSAI was 2.94 (SD = 0.83), the mean discrepancy score was 3.35 (SD = 0.89), and pretest intention was on average 4.52 (SD = 1.62). To check the randomization, the EMi conditions were compared on these five variables. The conditions did not differ significantly (*p*’s > 0.15) on all these variables, suggesting that the randomization was successful.

#### Effects on Negative Self-Evaluation

**FIGURE 4:**
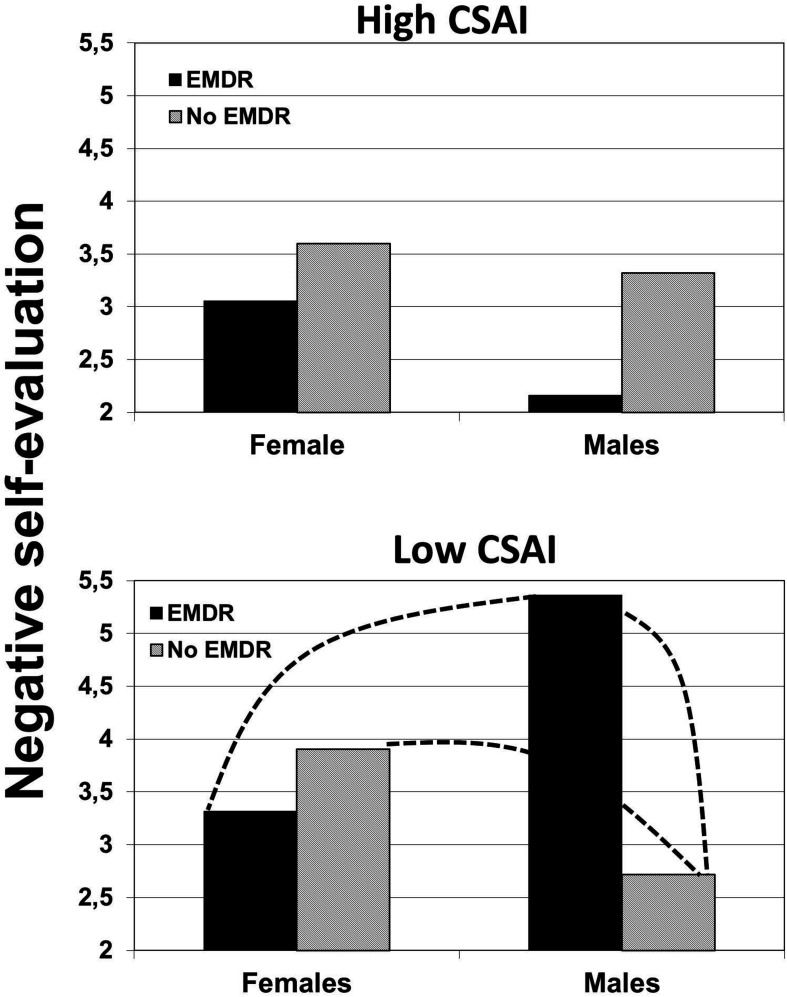
The effects of induced eye movements (EMi) on negative self-evaluation, moderated by CSAI and gender (the dotted line indicates a significant difference).

The same as in Study 1, a three-way interaction was conducted in a saturated model (with the three-way interaction as highest order factor), with pretest intention and pretest discrepancy as covariates, and negative self-evaluation as dependent variable, using an ANCOVA. This three-way interaction was significant, *F*(1,89) = 5.06, *p* < 0.027, η^2^ = 0.054. Figure 4 shows the estimated means of the effects of EMi on negative self-evaluation according to CSAI and gender.

To further understand the three-way interaction, it was tested whether the interaction between gender and EMi differed within each of the two levels of CSAI. Using the same method as explained above, two levels of CSAI were modeled. When CSAI was modeled as low, the gender × EMi interaction was significant, *F*(1,89) = 9.06, *p* = 0.003, η^2^ = 0.09. As can be observed in Figure 4, the expected effect of EMi occurred among males. To further test the pattern of means, the interaction between CSAI and EMi in only males was tested, which was also significant, *F*(1,34) = 7.0, *p* = 0.012, η^2^ = 0.19. Contrast analyses showed that in males, EMi led to a significant increase in negative self-evaluation (mean = 5.36) compared to no EMi (mean = 2.72), *p* = 0.004, diff. 95% CI = −4.42 to −0.87, η^2^ = 0.09, but also compared to females in the EMi condition (mean = 3.32), *p* = 0.018, diff. 95% CI = 0.36 to 3.74, η^2^ = 0.061. Lastly, within the no-EMi condition, males (mean = 2.72) showed a less negative self-evaluation compared to females (mean = 3.9), a difference that only approached significance, *p* = 0.073, diff. 95% CI = −2.48 to 0.11, η^2^ = 0.036. When CSAI was modeled as high, the gender × EMi interaction was not significant, *p* > 0.59, and no contrasts were significant. Thus, in Study 2, with participants recruited at another time through largely different Facebook pages only in the Netherlands, a very similar pattern of means was found as in Study 1.

## General Discussion

Defensive reactions toward health messages regarding fruit and vegetable consumption can lower the effectiveness of persuasion. The present study showed that induced eye movements can prevent or disturb defensive reactions, thereby restoring the messages’ potential to induce behavior change. The results not only replicate earlier findings (Dijkstra and Elbert, 2019) but also extend these by showing that the effects occur especially in males.

The two expected independent two-way interactions of EMi with CSAI and with gender at best approached significance, although all means were in the expected directions. Such a finding mostly brings up the question: “In whom will this interaction be more pronounced?” The three-way interaction gave the answer; it showed that CSAI and gender worked synergistically. Looking at all three figures, the low scores on negative self-evaluation and consumption of males with a low CSAI resemble a defensive reaction. In reaction to an auditory message, advocating fruit and vegetable consumption (and without EMi), especially men with lower CSAI scores, showed a less negative self-evaluation and lower consumption. The low score on negative self-evaluation suggests successful emotion regulation; despite the message implying that the recipient is endangering oneself, males who scored low on CSAI evaluated themselves as less negative, in contrast to females low on CSAI. In our theorizing, this low negative self-evaluation is caused by a mental image that is down-regulated in threat to repair the self-evaluation and avoid the aversive state of conflict (Dijkstra and Buunk, 2008). This mental image of lower quality also has less persuasive power to change behavior (as additionally observed from the low scores on consumption). In these males scoring low on CSAI, EMi led to a significant increase in negative self-evaluation and in fruit and vegetable consumption, EMi disturbed the defensive processes that undermined the quality, vividness, and emotionality, of the mental images.

When males scored high on CSAI (and without EMi), they had especially high scores on consumption. In our theorizing, this is caused by the positive and persuasion-supporting processes that are associated with high CSAI (Pietersma and Dijkstra, 2011a). In these males scoring high on CSAI, EMi led to a significant decrease in fruit and vegetable consumption (but not in negative self-evaluation), suggesting that the positive processes had been disturbed.

Whereas in males the effects of EMi significantly depended on CSAI, in females there was no trace of an interaction. Thus, although males and females can both score high or low on our measure of emotion-regulation CSAI, it seems to work differently for them. We must conclude that in females, CSAI was not related to any WM demanding processes that could be disturbed by EMi. There may be different reasons for that. CSAI is an individual difference that manifests only when a threshold of threat is passed. It may be that only in males the threat caused by the present auditory message passed a threshold and activated this emotion regulation. This might mean that females would do the same but only when the threat is higher. Moreover, it may also be that males’ threshold to engage in emotion regulation is lower. The present study cannot unravel this issue, but these results are in line with literature suggesting that males are more inclined to react negatively to persuasive stimuli, to use different emotion-regulation strategies, and to show more defensive reactions (Knight et al., 2002; Goldenbeld et al., 2008; McRae et al., 2008). The present study not only adds to the complex puzzle on gender differences in persuasion, but it also shows that gender might be used to match persuasive techniques (e.g., EMi) in practice.

It might be argued that the present male samples were (too) small (approximately 40). However, despite this smaller number of male participants, all three two-way interactions (CSAI × EMi) were significant, and the effect sizes (partial η^2^) of the specific contrast (EMi vs. no EMi in males with low CSAI) were above medium (0.066–0.09). Still, small sample size may even undermine claims based on significant tests (Button et al., 2013). Therefore, it is important to observe that the patterns of means were in the expected directions. We observed a full replication of the interaction between EMi and CSAI (Dijkstra and Elbert, 2019), but in the present study in males only. This replication on self-reported fruit and vegetable consumption is particularly relevant as the earlier study was conducted among university students who were exposed to EMi in a controlled environment in the laboratory. The present study was not conducted among students, and in an uncontrolled setting, wherever participants were when they listened to the audio. Thus, despite the error variance that must have occurred from this, the expected pattern of means on consumption was observed: When CSAI is low, EMi leads to more persuasion, but when CSAI is high, EMi lowers persuasion. Furthermore, Study 1 effects on negative self-evaluation were replicated in Study 2. These patterns are fully compatible with EMi taxing the WM, thereby preventing the dominant processes (negative or positive) to occur. Still, there is a need for an even more rigorous test of the present 3-way interaction, ideally conducted with a preregistered and fully powered study with balanced numbers of males and females.

One major uncertainty of the present study is the exposure to the manipulations: How well did the participants listen to the audio, and how well did participants comply with the eye movements assignment? Indeed, some participants admitted that they did not succeed in watching the stimulus all the time. In addition, although before the exposure the participants were asked twice to use a large screen, almost 40% admitted afterward to have used their smartphone. Although we do not know whether screen size is relevant for the effects of eye movements, when participants are not exposed correctly or sufficiently, the predicted effects may not come about. Therefore, on forehand, we chose to omit participants who scored below the midpoint on the self-reported exposure question. In addition, we tested whether screen size made a difference with regard to the expected effects. However, including screen as a variable in the computations hardly influenced the two-way interactions of EMi with CSAI and gender, and the main three-way interactions. Thus, the present study provides practical proof that despite not controlling the exposure completely, with the right instruction we were able to influence emotions and behavior change in expected directions.

This holds promise for EMi to be used in practice, for example, in online persuasion. One application is to match the EMi to those who can be predicted to react defensively. On the basis of the present study, the algorithm could be “If CSAI score is low AND gender is MALE, THEN apply EMi.” This would lead to the prevention of the inhibited scores on negative self-evaluation and the lowered scores on consumption, without the undesired effect of EMi in males who score high on CSAI. Besides having practical potential, EMi have a great potential as research tool to study all kinds of processes in the WM.

Some further limitations must be taken into account when interpreting the present study. Besides the non-optimal gender ratio and the limited insight into the adherence to our EMi manipulation, no direct measure of defensiveness was applied. Thus, we have to rely on interpretations of contextual variables and effects and have no direct sight on what happened during the processing of the persuasive information and EMi. Future studies on EMi might apply one or more different types of measures of defensiveness (Ruiter et al., 2001; Good and Abraham, 2007). Furthermore, one choice we made concerns the measurement of self-reported fruit and vegetable consumption: This variable was not assessed at pretest deliberately, because the follow-up measurement was only after 2 weeks, and we wanted to avoid people to remember their pretest score when they would self-report their behavior at follow-up. However, it is hard to argue how the present findings could be caused by the omission of the pretest measurement. In addition, the present analyses were all controlled for two variables that indicate the participants’ baseline perceptions regarding fruit and vegetable consumption: perceived discrepancy and the intention to increase one’s intake. Controlling for these ruled out that the results of the three-way interaction tests were caused by these individual differences. Moreover, perceived discrepancy correlated significantly with later fruit and vegetable consumption. In that, it may be regarded as a (weak) indicator of baseline consumption, which was controlled for. Another limitation to mention is the duration of the follow-up; it was only 2 weeks after the exposure to our manipulations. It may well be that the present effects will be waned after several months, and only small effects are left. However, our data do suggest that we can influence actual behavior, and in a practical application, our procedure might be used to reach large populations, making small effects relevant (Glasgow et al., 1999). An additional note is needed with regard to the unique female voice that presented the auditory persuasive message. It is possible that a male voice (Rodero et al., 2012) or a qualitatively different female voice leads to different reactions in recipients. Lastly, we did not assess individual differences in WM capacity, which might be related to gender or to CSAI (Barrett et al., 2004).

To conclude, the two studies make a plausible case that EMi can support persuasion to increase fruit and vegetable consumption. As EMi targets the WM, its effects depend on what is happening in the WM. The present study suggests that CSAI and gender predict what takes place in the WM. The WMAP provides new angles on persuasion, and it may help to make health education more effective, for example, in increasing fruit and vegetable consumption in the population.

## Data Availability Statement

The raw data supporting the conclusions of this article will be made available by the authors, without undue reservation.

## Ethics Statement

The studies involving human participants were reviewed and approved by Ethical Committee Psychology (ECP) of the Faculty of Behavioral and Social Sciences. The patients/participants provided their written informed consent to participate in this study.

## Author Contributions

AD and SE contributed to the theorizing and preparation of the research, developing the research line using auditory messages, and writing the present manuscript. AD conducted the statistical analyses. Both authors contributed to the article and approved the submitted version.

## Conflict of Interest

The authors declare that the research was conducted in the absence of any commercial or financial relationships that could be construed as a potential conflict of interest.
